# Ribosome stalling-induced *NIP5;1* mRNA decay triggers ARGONAUTE1-dependent transcription downregulation

**DOI:** 10.1093/nar/gkaf159

**Published:** 2025-03-20

**Authors:** Mayuki Tanaka, Naoyuki Sotta, Susan Duncan, Yukako Chiba, Hitoshi Onouchi, Athanasius F M Marée, Satoshi Naito, Verônica A Grieneisen, Toru Fujiwara

**Affiliations:** Graduate School of Agricultural and Life Sciences, The University of Tokyo, Tokyo 113-8657, Japan; Graduate School of Agriculture, Osaka Metropolitan University, Osaka 599-8531, Japan; Graduate School of Agricultural and Life Sciences, The University of Tokyo, Tokyo 113-8657, Japan; Graduate School of Agriculture, Osaka Metropolitan University, Osaka 599-8531, Japan; School of Biosciences, Cardiff University, Cardiff CF10 3AX, United Kingdom; Department of Cell and Developmental Biology, John Innes Centre, Norwich Research Park, Norwich NR4 7UH, United Kingdom; Graduate School of Life Science, Hokkaido University, Sapporo 060-0810, Japan; Faculty of Science, Hokkaido University, Sapporo 060-0810, Japan; Graduate School of Agriculture, Hokkaido University, Sapporo 060-8589, Japan; School of Biosciences, Cardiff University, Cardiff CF10 3AX, United Kingdom; Graduate School of Life Science, Hokkaido University, Sapporo 060-0810, Japan; Graduate School of Agriculture, Hokkaido University, Sapporo 060-8589, Japan; School of Biosciences, Cardiff University, Cardiff CF10 3AX, United Kingdom; Graduate School of Agricultural and Life Sciences, The University of Tokyo, Tokyo 113-8657, Japan

## Abstract

In eukaryotes, messenger RNA (mRNA) accumulation is regulated through the levels of transcription, processing, and degradation. Here, we uncover the multi-level regulatory mechanism governing the expression of *NIP5;1*, a boron (B) diffusion facilitator in *Arabidopsis*. B-dependent *NIP5;1* mRNA degradation is triggered by ribosome stalling at an AUGUAA sequence in its 5′-untranslated region. We showed that deletion of ATGTAA also abolishes B-dependent transcriptional downregulation, revealing a dual role of this sequence in both mRNA degradation and transcriptional control. Small RNAs (sRNAs) and ARGONAUTE1 (AGO1) are implicated in mRNA-degradation-mediated B-dependent transcriptional downregulation: a 5′–3′ exonuclease mutant, *xrn4*, presents both elevated levels of *NIP5;1* mRNA degradation intermediates and transcriptional downregulation; AGO1-associated sRNA-sequencing reveals the presence of sRNAs with sequences upstream of *NIP5;1* AUGUAA; and nascent mRNA profiling by global run-on sequencing demonstrates RNA polymerase II pausing at ATGTAA, a phenomenon diminished in the *ago1* mutant that lacks B-dependent transcriptional downregulation. These findings point to multi-level coordination of *NIP5;1* expression with the AUGUAA sequence at its core: ribosome stalling orchestrates translational inhibition, mRNA degradation and transcriptional downregulation in response to B. The fast response resulting from this synergy suggests that similar mechanisms may exist in other eukaryotic systems for efficient and rapid regulation of gene expression.

## Graphical Abstract

**Graphical Abstract:**
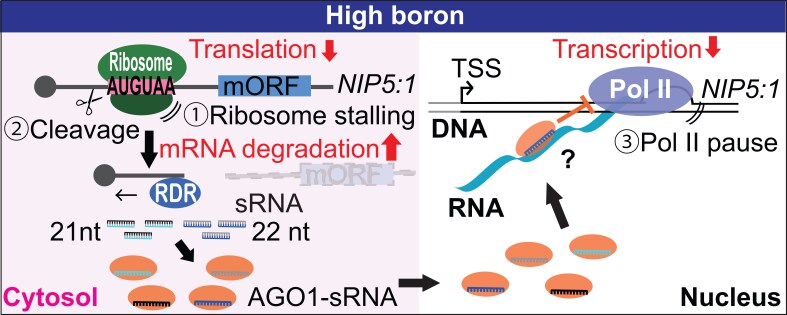

## Introduction

In eukaryotes, gene expression involves multiple processes that take place in different cellular compartments. After DNA transcription in the nucleus, pre-messenger RNA (mRNA) undergoes post-transcriptional modification, such as capping, splicing, 3′ end truncation and poly(A) addition, giving rise to mature mRNA that is transported to the cytoplasm by nuclear export factors and subjected to translation. Stability of mRNA in the cytoplasm greatly contributes to the resulting protein levels [1–4]. In many cases mRNA stability is mediated by the 5′- or 3′-untranslated region (UTR) of the mRNA, for example, through the upstream open reading frames (uORFs) that are present on the 5′-UTR. In certain cases, uORFs are able to trigger mRNA degradation by inducing ribosome stalling [5–10]. Importantly, besides triggering mRNA degradation, ribosome stalling impedes processivity and this ultimately reduces translation of the main ORF [8, 11–13].

Molecular mechanisms that regulate transcription in the nucleus and mRNA degradation in the cytoplasm were initially investigated independently. However emerging evidence indicates that intricate feedback exists between synthesis and degradation pathways to regulate overall transcript levels [14]. To date, studies on zebrafish and mouse indicate that mRNA degradation induced by a premature stop codon (nonsense-mediated mRNA decay; NMD) can act as a trigger for transcriptional upregulation of genes that are functionally related to the modified gene [15, 16]. A model has been proposed in which degraded mRNA fragments of the modified gene are transported from the cytosol into the nucleus to upregulate transcription through chromatin modification. This provides a potential mechanistic explanation for the phenomenon known as the genetic compensation response, in which detrimental effects of some mutations can be compensated by upregulation of functionally related genes [17].

Interactions between mRNA degradation and transcription have also been demonstrated in yeast. Mutations in factors involved in mRNA decay, including the cytoplasmic 5′–3′ exoribonuclease XRN1p, the decapping factor Dcp2, and the deadenylation factor Not1, stabilize the mRNA of various genes but do not increase their steady-state mRNA accumulation [18, 19]. These results suggest that changes in mRNA half-life are compensated by inverse changes in transcription. A circular-regulation model based on the mRNA life cycle has been proposed to explain this compensation: mRNA decay factors would not only degrade uncapped mRNA but would also be imported into the nucleus to stimulate transcription, conferring robustness of proper mRNA levels [20]. An image-based genome-wide screen has also indicated that nuclear mRNA concentrations negatively regulate RNA polymerase II (Pol II) activity and abundance in human cells [21]. In addition to being central to gene expression homeostasis, there is also evidence of feedback between mRNA degradation and transcription activity in response to viral infection in human cells. In this scenario, Xrn1-dependent mRNA decay can suppress transcription through translocation of RNA binding proteins to the nucleus [22]. In their model, the cytoplasmic poly(A) binding protein is relocalized to the nucleus after mRNA degradation, repressing recruitment of TATA binding protein and Pol II to the promoter regions.

Evidence of conserved mRNA concentration homeostasis has been reported for *Arabidopsis* [23, 24] Furthermore, although a mutation in the 3′–5′ exoribonuclease Suppressor Of Varicose (SOV) altered mRNA stability for many genes, overall mRNA levels were maintained [25]. However, very little is known about the underlying feedback between mRNA production and degradation pathways that govern this conserved homeostatic system in plants. Their sessile nature, however, suggests that multi-level, interconnected mRNA regulation has evolved to support plant survival under dynamic environmental conditions.

Boron (B) is an essential nutrient for plants but is toxic when present in excess [26, 27]. NIP5;1 is a boric acid transporter that facilitates B absorption from the soil under limited B conditions [28]. *NIP5;1* mRNA accumulation is increased under low B conditions, enhancing B absorption, whereas it is destabilized under high B conditions, to prevent excessive B uptake. B-dependent *NIP5;1* mRNA degradation is triggered by ribosome stalling on the AUGUAA sequence in the 5′-UTR [8]. B then hampers its re-initiation for downstream translation and stabilizes the translation termination factor, eRF1, which binds to the A site of the stalled ribosome. This stabilization facilitates the interaction between eRF1 and Met-tRNA_i,_ which binds to the P site of the ribosome, promoting the hydrolysis of methionine. This process leads to temporary ribosome stalling, whereby mRNA is cleaved and degraded [29]. This mechanism yields B-dependent gene expression through AUGUAA at two levels: mRNA accumulation and translation efficiency [8]. A recent study has shown that *NIP5;1* expression involves STOP1, a transcription factor that regulates various genes in multiple stress responses [30]. However, the expression of STOP1 does not respond to B conditions, and *in vivo* evidence for B-dependent transcriptional regulation of *NIP5;1* has not yet been provided.

Here, with detailed characterization of *NIP5;1* mRNA response to different B conditions, we show that *NIP5;1* mRNA degradation by itself cannot fully explain *NIP5;1* mRNA accumulation when responding to varying B conditions. Instead, *NIP5;1* mRNA accumulation is regulated not only by mRNA degradation but also by transcriptional control, with both processes requiring the AUGUAA (on mRNA)/ATGTAA (on DNA) sequence. Furthermore, we found that AGO1 and mRNA degradation intermediates are involved in the transcriptional downregulation. Our study demonstrates a regulation model in which the translation process not only senses nutrient status to selectively degrade mRNA and reduce translation, but the resultant mRNA degradation process is also able to function, in combination with AGO1, as a trigger for transcription downregulation. The manner in which these processes are intertwined generates a multi-level coupling between transcription, mRNA degradation, and translation, yielding a high level of control and swift response to changing conditions.

## Materials and methods

### Plant materials and growth conditions

**Figure 1.**
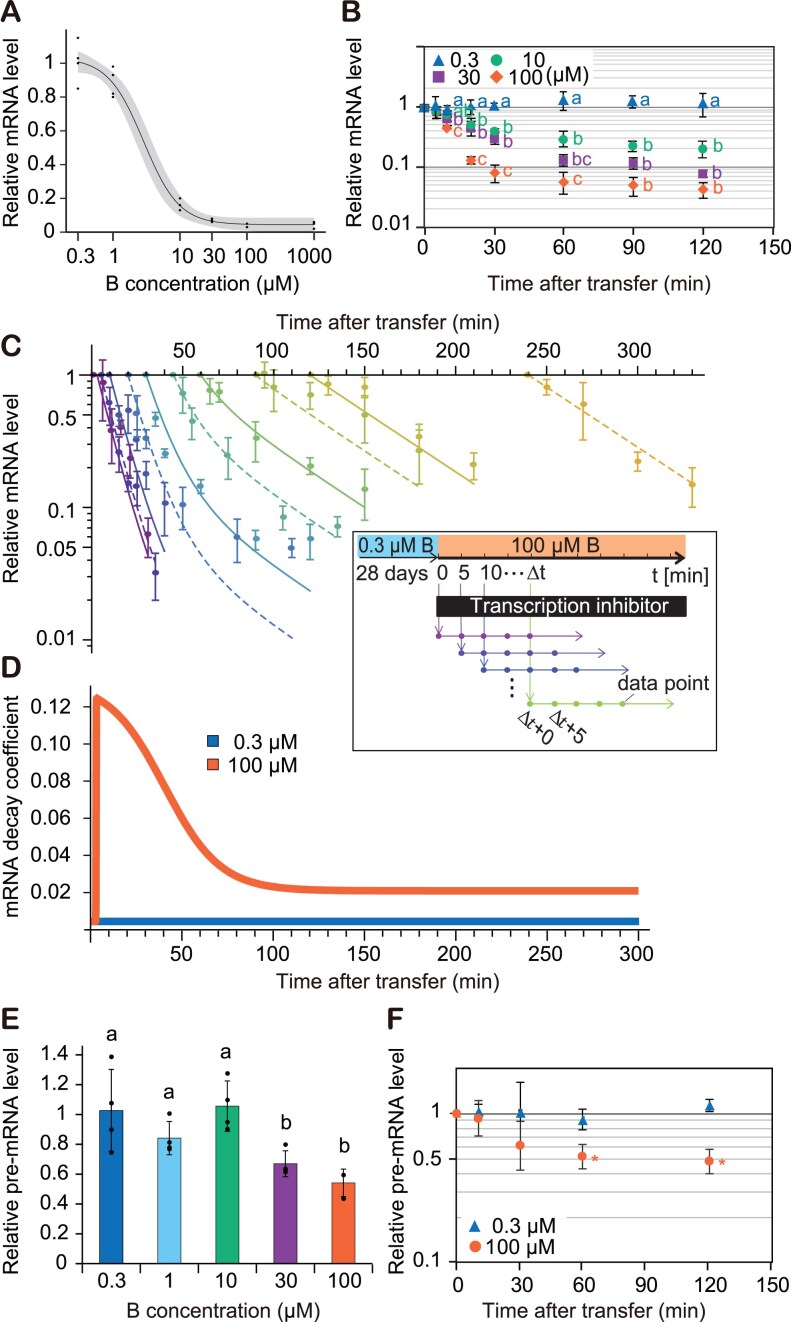
B-dependent mRNA degradation and transcription of *NIP5;1*. (**A**) Steady-state levels of *NIP5;1* mRNA accumulation in roots grown under various B conditions for 10 d (*n* = 3 biological replicates). Black line and gray shading represent logit regression with the 95% confidence interval. (**B**) Time-course measurements of mRNA levels following shifts to various B conditions after 28-d preculture under 0.3 μM B (*n* = 4 biological replicates). (**C**) Time-course measurement of mRNA degradation after a shift from 0.3 to 100 μM B. Colours of data points represent datasets with different timings of cordycepin application. Lines are the fitted curves from the time-dependent degradation model. [panel (C), inset] Experimental design of the mRNA degradation time-course measurements. Transcription inhibitor (cordycepin) was added Δt min after a shift from 0.3 to 100 μM B. Roots were sampled for RNA extraction at several time points after cordycepin addition. (**D**) mRNA degradation rate curves obtained by the model fitting. (**E**, **F**) *NIP5;1* pre-mRNA accumulation as an indicator of transcription activity. Seedlings precultured with 0.3 μM B for 28 d were used. (**E**) Steady-state pre-mRNA levels relative to the level under the 0.3 μM B condition were measured after 2 h treatment with various concentrations of B. (**F**) Time-course measurements of pre-mRNA changes after a shift to 100 μM B. Asterisks indicate significant difference from before the shift (0 min) at *P* < .05 using the Dunnett’s test. Groups sharing the same alphabets are not significantly different at *P* < .05 using the Tukey–Kramer’s test. For panel (B), independent statistical tests were performed for each individual time point. Shown are means ± standard deviation (SD) of relative (pre-)mRNA accumulation (*n* = 3–4 biological replicates).

**Figure 2.**
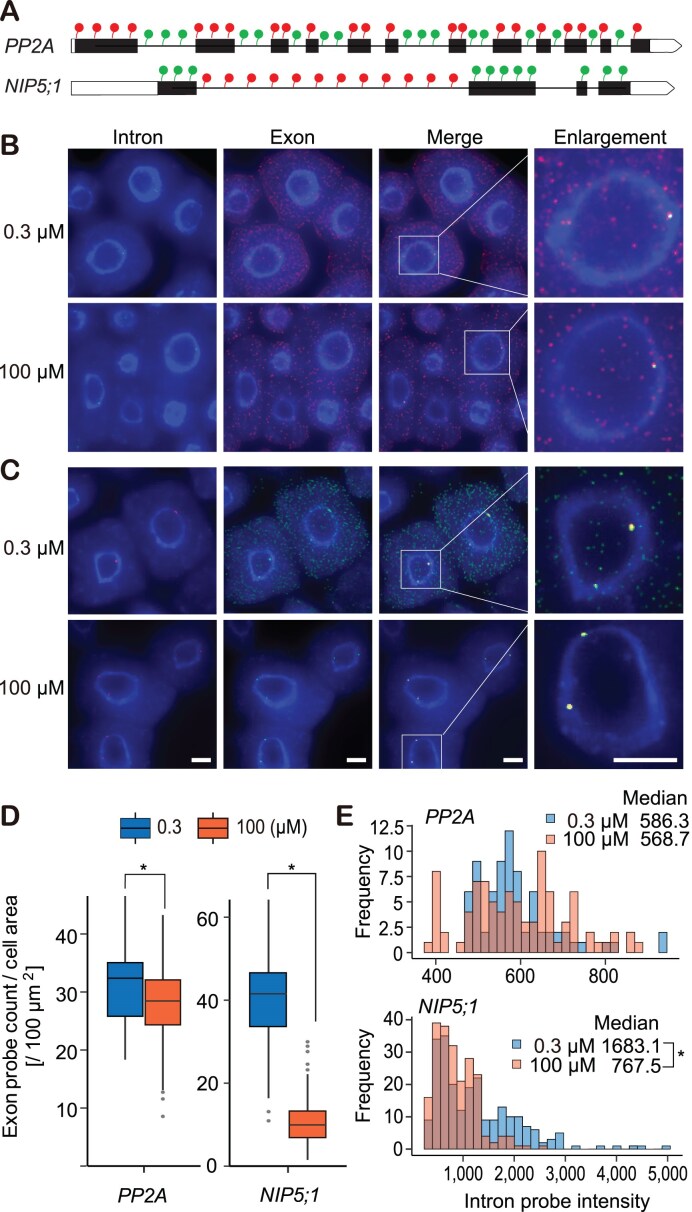
B-dependent mRNA accumulation and transcription activity at the single cell level. (**A**) Probe design for single-molecule fluorescence *in situ* hybridization (smFISH). Exons (filled boxes, coding regions; open boxes, UTRs) and introns (lines) were labeled with different fluorescent dyes. (**B**, **C**) Representative smFISH images of *PP2A* intron (green) versus exon (red) (**B**) and *NIP5;1* intron (red) versus exon (green) (**C**). Plants were grown under 0.3 or 100 μM B for 5 d. DNA was stained with 4′,6-Diamidino-2-phenylindole (blue). Scale bar, 5 μm. (**D**) Quantification of exon probe density in cells. Box plots represent median and quantiles of exon probe count per cell area of *PP2A* (*n* = 55, 0.3 μM B; *n* = 49, 100 μM B) and *NIP5;1* (*n* = 120, 0.3 μM B; *n* = 119, 100 μM B). (**E**) Quantification of intron probe intensity at transcription sites. Histograms represent distribution of intron probe intensity of *PP2A* (*n* = 91, 0.3 μM B; *n* = 74, 100 μM B) and *NIP5;1* (*n* = 231, 0.3 μM B; *n* = 214, 100 μM B). Asterisks indicate significant difference at *P* < .05 using the Willcoxon rank sum test.

*Arabidopsis thaliana* (L.) Heynh. ecotype Columbia (Col-0) was used as a wild-type plant in this study. *nip5;1–1* (Salk_122287), *xrn4-5* (SAIL_681_E01), and *xrn4-6* (Salk_014209) were described previously [28, 31]. *ago1-27* is an ethyl methane sulfonate mutant line in which alanine-992 is substituted by valine [32]. *rdr1/2/6* is triple mutant of *rdr1-1*, *rdr2-1*, and *rdr6-15* [33]. *rrp44aKD-1* and *rrp41KD-1* are knockdown lines generated using an artificial microRNA strategy [34]. It should be noted that Col-0 has a mutation in *SOV/DIS3L2* which is a 3′–5′ exoribonuclease. *SOV/DIS3L2* has a genetic interaction with *XRN4*, which may affect *NIP5;1* B-dependent mRNA degradation in Col-0 [25]. Transgenic plants 5′*NIP5;1^WT^:GUS*, 5′*NIP5;1^WT^:NIP5;1*, and 5′*NIP5;1^ΔATGTAA^:NIP5;1* have been described previously [8, 35] referred to as *P_-2, 180UTR312_-GUS*, *ProNIP5;1:5*′*-NIP5;1(-558):NIP5;1*^-139^UAUA/^-122^AUGUAA, and *ProNIP5;1:5*′*-NIP5;1(-558):NIP5;1*^-139^UAUA/^-122^AUGUAA*^Δ^*, respectively. Crossing the *nip5;1–1* mutant plant carrying 5′*NIP5;1^WT^:NIP5;1* with a plant carrying 5′*NIP5;1^WT^:GUS* generated a double homozygous line in the F_3_ generation. Except for Figs 1A and 2, plants were grown on solid medium [36] containing 1% (w/v) sucrose with 0.15% (w/v) gellan gum (Wako Pure Chemicals) at 22°C under long-day conditions (16 h light/8 h dark cycle). For Figs 1A and 2, plants were grown on hydroponic culture medium containing 1% (w/v) sucrose, and solidified with 1% (w/v) gellan gum at 22°C under long-day conditions. At least three independent biological replicates were examined in all experiments.

### Quantification of transcript accumulation by qRT-PCR

Except for the data shown in Figs 1A, 4C, and 5E, plants were grown under 0.3 μM B for 27 d and transferred to hydroponic culture medium containing the same concentration of B for 1 d. Plants were then transferred to media containing various B conditions for 2 h. For the data shown in Figs 4C and 5E, plants were grown under 10 μM B for 27 d and were transferred to media containing 0.3 μM B conditions for 1 d and were then transferred to media containing 0.3 and 100 μM B conditions for 2 h. For the data shown in Fig. 1A, plants were grown under various B conditions for 10 d. Total RNA from root samples was extracted using a NucleoSpin RNA Plant kit (MACHEREY-NAGEL). For mRNA quantification, 500 ng of total RNA was reverse-transcribed in a 10 μl reaction mixture using a PrimeScript RT reagent kit (Takara Bio). For pre-mRNA quantification, 1 μg of total RNA was subjected to genome DNA depletion with genomic DNA Eraser (Takara Bio) in a 10 μl scale. A 5 μl reaction mixture was then reverse-transcribed using a PrimeScript RT Master Mix; (Takara Bio) in a 10 μl reaction mixture with a *NIP5;1*-specific primer (5′-ATCATCACTGCGAGTCCTGC-3′). The other 5 μl reaction was used for the non- reverse transcription (non- RT) control that was treated in the same way as the other half except for the addition of reverse transcriptase. The complementary DNA was quantified by real-time polymerase chain reaction (PCR) using a Thermal Cycler Dice Real Time System TP800 (Takara Bio) with a SYBR Premix Ex Taq kit (Takara Bio). We used two reference genes (*eEF1α*, *Actin10*) in the initial phase of the experiments. Since similar data were obtained with the two genes, we used *eEF1α* as a reference gene for subsequent analyses. The following primers were used: for *NIP5;1* mRNA, forward, 5′-CACCGATTTTCCCTCTCCTGAT-3′ and reverse, 5′-GCATGCAGCGTTACCGATTA-3′, for *NIP5;1* pre mRNA, forward, 5′-CTCTTTCTTACTCTCTAGCCTC-3′ and reverse, 5′- GAATGTTCCCACGAACTCGG-3′, for e*EF1α* mRNA, forward, 5′-CCTTGGTGTCAAGCAGATGA-3′ and reverse, 5′-TGAAGACACCTCCTTGATGATTT-3′. At least two biological replicates were included in quantitative RT-PCR (qRT-PCR) reactions for each sample.

### mRNA half-life measurements

mRNA and pre-mRNA half-lives were determined as described previously [35]. Briefly, for assessing the time-dependent *NIP5:1* mRNA degradation shown in Fig. 1C and Supplementary Fig. S1, plants were grown on solid medium containing 0.3 μM B for 27 d and transferred to hydroponic culture medium containing the same concentration of B for 1 d. Plants were then transferred to hydroponic culture medium containing 0.3 or 100 μM B for periods varying between 1 min and 24 h. Following pre-incubation, 3′-deoxyadenosine (cordycepin) (Funakoshi) was added to the medium at a final concentration of 0.6 mM (*t* = 0 min), and immediately vacuum-infiltrated for 45 s. Root samples were harvested at four or five time points between 0 and 240 min and frozen in liquid nitrogen. For the pre-mRNA degradation measurements in Supplementary Fig. S2, plants were grown on solid medium containing 0.3 μM or 100 μM B for 27 d and transferred to hydroponic culture medium containing the same concentration of B for 1 d. Cordycepin was then added to the medium and root samples were harvested at 0, 10, 30, and 60 min. Total RNA was isolated and analyzed by qRT-PCR. mRNA accumulation relative to the time point of cordycepin application, taken as *t* = 0, was determined at each time point. The mRNA half-lives were calculated by linear regression using the least-square method on log-converted relative mRNA accumulation over time. At least two technical replicates were included in qRT-PCR reactions for each sample.

### Modelling of mRNA dynamics

mRNA concentration determined by qRT-PCR was normalized relative to the value at time 0, the moment of transcription inhibitor application, for each series. Against these values, the following differential equation for mRNA dynamics was applied:


\begin{equation*}\frac{{dA}}{{dt}} = k - \lambda A\end{equation*}

where *A* is the normalized mRNA concentration, *k* is the transcription rate, and λ is the degradation rate. Given that the dynamics were captured after application of the transcription inhibitor, *k* was assumed to be negligible, and hence set to 0. For λ, we tested two different models, one in which λ is assumed to be constant over time, and another in which λ is allowed to vary over time. We use the ‘impulse model’ to capture the time-dependency of degradation, which is a six-parameter double sigmoidal function [37]. Parameter fitting was performed using Mathematica 11.3. The equation was numerically solved for each parameter set using the NDSolve function. The FindMinimum function was then utilized to minimize the relative error between the model and the experimental values. During this fitting, λ at *t* = 0 was set to the level found for the pre-treatment condition (0.3 μM B), obtained from the constant model. For model selection, Akaike'sinformation criterion with small-sample adjustment and probability was calculated as described in Marée *et al.* [38].

### smFISH analysis

Plants were grown under 0.3 and 100 μM B conditions for 5 d. smFISH was performed as previously described [39, 40]. Image acquisition was conducted using a Zeiss Elyra PS1 inverted microscope with laser settings and filters as specified in [41]. mRNA molecules were quantified using the automated image-processing program FISHcount [42]. To analyze intron probe intensities, Fiji [43] was used with a custom macro. Z-stack images from the probe channel were projected using the ‘Average Intensity’ method, followed by background subtraction using the ‘Subtract Background’ function (rolling ball radius = 20). Transcription sites were detected through binarization with auto-thresholding (‘MaxEntropy’ mode), and further analyzed using the ‘Analyze Particles’ function (size range = 2–200 pixels). Mean pixel intensities of each detected region of interest were recorded. Artifactual signals, such as those detected outside nuclei, were identified via manual inspection and excluded from the analysis.

### Primer extension analysis

Primer extension analysis of mRNA shown in Fig. 4B was performed using poly(A) RNA extracted from *Arabidopsis* plants as described previously [8]. Briefly, plants were grown on solid medium containing 0.3 μM B for 28 d and transferred to hydroponic culture medium containing 0.3 μM B for 2 h (−B) and 100 μM B for 2 h (+B) and 10 min (T). The following primer was used: 5′-TCGAGGCGTTGGTTTCCGATGATC-3′. Poly(A) RNA (500 ng) was annealed with the ^32^P-labeled primer in 40 μl of annealing buffer at 58°C for 2 h, and the reverse transcription reaction was carried out using Thermoscript RNase H^−^ reverse transcriptase (Invitrogen) at 58°C for 60 min. Following phenol-chloroform extraction, the samples were separated on a 6% polyacrylamide/7 M urea gel. DNA sequence ladders were prepared using the DNA Cycle Sequencing System (Promega) by using the same primer and RNA construct carrying the AUGUAA sequence as a template.

### Reanalysis of the global run-on sequencing, AGO1-associated nucleic small RNA-seq, degradome, and CAGE-seq public datasets

FASTQ files for AGO1-associated nucleic small RNA-seq (sRNA-seq), global run-on sequencing (GRO-seq) [44], CAGE-seq [45], and degradome-seq [46] were downloaded from NCBI using SRA Tools (https://github.com/ncbi/sra-tools) or wget. Accession numbers of the samples used in the reanalysis are summarized in Supplementary Table S1. Reads were pre-processed with the FASTX-Toolkit (https://github.com/agordon/fastx_toolkit) and Trim Galore [47]. Low quality reads were removed by ‘fastq_quality_filter -q 20 -p 85’. Adopter sequences were removed by ‘fastx_clipper -a TCGTATGCCGTCTTCTGCTTG | fastx_clipper -l 32 -a AAAAAAAAAAAAAAAAAAAA’ for GRO-seq, ‘trim_galore -a AGATCGGAAGAGCACACGTCTGAACTCCAGTCAC’ for sRNA-seq, ‘trim_galore -q 20 –length 30’ for CAGE-seq, and ‘trim_galore -q 20 –length 12 -a TGGAATTCTCGG’ for degradome. Reads derived from ribosomal RNA (rRNA) and other noncoding RNAs were removed by mapping the reads to reference sequences consisting of rRNAs, transfer RNAs, and noncoding RNAs by using bowtie2 [48] (version 2.3.5) with the ‘–un’ option. The pre-processed reads were mapped to the TAIR10 genome reference by bowtie2 (for GRO-seq) or STAR [49] (for sRNA-seq, CAGE-seq, and degradome). Uniquely mapped reads with mapping quality no <10 were used for the downstream analysis. Plotting profiles and read counting were done with R (version 3.5.1) using the Gviz and Rsamtools packages.

### 3′ rapid amplification of cDNA ends (3' RACE)

3′ RNA ligase-mediated RACE was performed as previously described [50, 51] with modifications. Wild-type (Col-0) plants were grown under 0.3 μM or 100 μM B for 21 d. For the sample in lane T, wild-type, *rrp44aKD-1*, and *rrp41KD-1* plants grown under 0.3 μM B for 21 d were transferred to hydroponic medium containing 100 μM B for 10 min. Total RNA (7 μg) prepared from roots was first 3′-dephosphorylated using T4 Polynucleotide Kinase (25 U) (NEB) in 10× Protruding End Kinase Buffer and Ribonuclease Inhibitor (20 U) (RNasin, Promega) for 1 h at 37 °C without ATP in a 10 μl scale. The enzyme was inactivated by heating at 75 °C for 10 min. Dephosphorylated RNA was then purified using ISOGEN (Nippongene) and resuspended in 6 μl of RNase-free water. RNA was denatured at 80 °C for 5 min before linker ligation. The ligation reaction in a 20 μl scale included dephosphorylated, denatured RNA, T4 RNA Ligase 2 (200 U), truncated K227Q (NEB), 10× T4 RNA Ligase 2 buffer, Ribonuclease Inhibitor (40 U), 50% polyethylene glycol, and 100 μM pre-adenylated linker (Universal miRNA Cloning Linker, NEB). The reaction was incubated at 22 °C for 2 h, followed by 25 °C for 2 h. Ligated RNA was purified using ISOGEN and resuspended in 10 μl of RNase-free water. Reverse transcription in a 20 μl scale was performed using the ligated RNA sample (10 μl) with SuperScript III (Invitrogen) and the Universal Linker R primer (5′-ATTGATGGTGCCTACAG-3′), complementary to the Universal miRNA Cloning Linker sequence. PCR amplification was conducted using the NIP5;1-TSS primer (5′-TAAGCTCAAAGACTAACCAAACCCA-3′), specific to the transcription start site of *NIP5;1*, and Universal Linker R. The PCR products were separated on a 1.5% agarose gel. Around 250 bp band was excised and sequenced.

### Statistical analyses

All data in graphs are presented as the mean ± SD. Statistical analysis was performed using a Tukey–Kramer’s test, Dunnett’s test, Willcoxon rank sum test, or Student’s *t*-test. Significant differences were defined as a *P*-value < .05.

### Accession numbers

*Arabidopsis* sequence data from this article can be found in the Arabidopsis Genome Initiative and GenBank/EMBL/DDBJ databases under the following accession numbers: NIP5;1, At4g10380; AGO1, At1g48410; RDR1, At1g14790; RDR2, At4g11130; RDR6, At3g49500; RRP44A, At2g17510; RRP41, At3g61620; XRN4, At1g54490.

## Results

### *NIP5;1* mRNA downregulation occurs in a B dose-sensitive manner

Previous studies have shown that *NIP5;1* mRNA accumulates ∼25-fold more in plants grown under B-deficient (0.3 μM B) conditions compared to B-sufficient (100 μM B) conditions, which has been linked to increased mRNA degradation in the presence of sufficient B [35]. To better understand the regulation of *NIP5;1* in response to B, we first examined how differences in B concentrations alter *NIP5;1* mRNA accumulation. To quantify *NIP5;1* mRNA accumulation in response to different B conditions, we measured steady-state levels of *NIP5;1* mRNA in *Arabidopsis* roots under 0.3, 1, 10, 30, 100, and 1000 μM B conditions (Fig. 1A). We calculated that *NIP5;1* mRNA accumulation at 10 μM B was only 16% of the level observed at 0.3 μM, and reduced to only 4.0% at 100 μM. mRNA accumulation at 1000 μM was not significantly different from the level detected at 100 μM, suggesting that B-dependent *NIP5;1* mRNA downregulation saturates between 30 and 100 μM B.

Next, we examined the dynamics of mRNA downregulation by transferring plants from B-deficient conditions to a range of B conditions. Seedlings grown under 0.3 μM B were exposed to 0.3, 10, 30, and 100 μM B, and *NIP5;1* mRNA accumulation levels were measured over time (Fig. 1B). Except for the 0.3–0.3 μM shift (i.e. a transfer in which the B conditions remain the same), a decrease in *NIP5;1* mRNA accumulation could be observed as early as 5 min after the change in B concentration, with a new steady-state being reached after around 90 min. Both the initial negative slope of the response curve and the final steady-state level were dependent on the B concentration, indicating that a dose-sensitive mechanism underlies *NIP5;1* mRNA downregulation in response to B (Fig. 1A and B).

### mRNA degradation alone cannot explain observed *NIP5;1* mRNA dynamics

Previous studies have revealed that *NIP5;1* mRNA downregulation involves B-dependent mRNA degradation [8, 35]. However, it remained unclear whether uORF-triggered mRNA degradation is the sole mechanism for the downregulation of B-dependent *NIP5;1* mRNA accumulation. To explore if this is the case, seedlings were pre-grown at 0.3 μM B and then transferred to either 0.3 or 100 μM B. Cordycepin, a transcription inhibitor, was then applied at time points ranging from 1 min to 24 h after the transfer, and root samples were collected at 0–120 min after cordycepin application (Fig. 1C and Supplementary Fig. S1). By tracking the decline in mRNA levels over time for different time points at which transcription inhibition is initiated, we determined the time-dependency of the mRNA degradation rate (Supplementary Fig. S1).

To characterize the temporal dynamics of mRNA degradation, we determined through model fitting the mRNA decay rate (λ) as a function of time after treatment and B concentration. We assumed first-order kinetics for mRNA degradation and fitted the data to two different models. The first, the ‘constant degradation model’, assumes that λ is constant over time; while the second, the ‘time-dependent degradation model’, expresses λ as a double sigmoidal function to capture temporal changes in the degradation rate [37]. We then performed model selection using Akaike’s information criterion with small-sample adjustment [52]. This analysis showed that the time-dependent degradation model is the best model to capture the 0.3–100 μM shift dataset, whereas the constant degradation model best captures the 0.3–0.3 μM nonshift control dataset (Fig. 1D and Supplementary Fig. S1). The results show that, while plant transfer alone does not alter underlying decay rates, shifting B conditions causes temporal changes in the *NIP5;1* mRNA degradation rate. The obtained function shows a transient response in the degradation rate to the shift in B conditions. In contrast to the degradation rate of 0.0044 min^−1^ (half-life = 158 min) without the shift, the degradation rate exhibits a single peak over time of 0.12 min^−1^ (half-life = 5.8 min), a 28-fold increase, at 5 min after the shift to 100 μM B, but decreases thereafter, until reaching a steady-state level of 0.021 min^−1^ (half-life = 33 min), a 4.7-fold increase, at ∼120 min after the shift (Fig. 1D).

Given that steady-state mRNA levels are determined by the ratio of transcription to mRNA degradation, transcription rates at steady-state can conversely be estimated from mRNA accumulation and degradation rates. At steady-state, i.e. 120 min after transfer, the degradation rate at 0.3 μM B is 21% of the rate at 100 μM, while the mRNA accumulation is 25-fold higher than at 100 μM B (compare Fig. 1D versus Fig. 1B). From this, it follows that the transcription rate at 0.3 μM B must be 5.3-fold higher than at 100 μM B. This result implies that increased B levels not only activate mRNA degradation, but also trigger downregulation of *NIP5;1* transcription.

### *NIP5;1* mRNA transcription is downregulated in response to B

To explore transcriptional regulation of *NIP5;1* in response to B, we monitored transcription rates by assessing *NIP5;1* nascent RNA. Pre-mRNA levels reflect transcription activity if residence time in the nucleus is not altered by the treatment of interest, for example, through changes in post-transcriptional modification rates [53]. To determine changes in pre-mRNA residence time, we first measured *NIP5;1* pre-mRNA half-life at steady state in the presence of cordycepin. Since we found that differing B conditions did not significantly affect pre-mRNA stability (half-life = 41 ± 7 min at 0.3 μM, 49 ± 9 min at 100 μM) (Supplementary Fig. S2), our results indicate that pre-mRNA levels can provide an accurate measure of *NIP5;1* transcription rate [54]. Next, we measured *NIP5;1* pre-mRNA accumulation under various B conditions. Compared to 0.3 μM B, *NIP5;1* pre-mRNA accumulation was reduced to 62% at 30 μM B, indicating that higher B levels suppress *NIP5;1* transcription. No further decrease was observed at 100 μM (Fig. 1E).

Data from our time-course experiment revealed a reduction in *NIP5;1* pre-mRNA levels ∼30 min after plants were transferred from 0.3 μM to 100 μM B. Pre-mRNA accumulation levelled off at 48% after 60 mins and reached a steady-state at around 120 min (Fig. 1F). Considering the *NIP5;1* pre-mRNA half-life was measured to be 49 ± 9 min when plants are exposed to 100 μM B (Supplementary Fig. S2), this suggests that transcriptional downregulation must have been activated no later than 10 min after plants are transferred to high B conditions.

To further investigate B mediated downregulation of *NIP5;1* transcription, we performed smFISH on root meristem cells [42] (Fig. 2). By utilizing intron- and exon-specific probes, smFISH was used to infer *NIP5;1* transcriptional activity and quantify *NIP5;1* mRNA molecules per cell, respectively, for seedlings grown under 0.3 or 100 μM B conditions. First, we used exon probes (Fig. 2A) to explore *NIP5;1* mRNA downregulation at the single cell level together with exons probes complimentary to a non-B-responding smFISH control gene *PP2A* [23]. As expected, although the number of exon probe signals detected for the control gene *PP2A* (Fig. 2A) were similar under 100 μM B and 0.3 μM conditions (91% of the level at 0.3 μM), this increase in B concentration resulted in a marked reduction in *NIP5;1* mRNA molecules (29% of the level at 0.3 μM) (Fig. 2B–D). These results demonstrate smFISH as an effective technique to explore *NIP5;1* mRNA downregulation at single cell resolution.

Next, we investigated transcriptional activity using probes designed to be complimentary to *NIP5;1* RNA introns. Consistent with high specificity, this probe set only generated signals in the nuclei. Typically, we observed 1–4 large *NIP5;1* intron spots that are indicative of transcriptional activity for this gene at the time of fixation [55, 56]. Given that the fluorescence intensity generated by intron probes typically reflects the quantity of nascent pre-mRNA at each site of transcription, we were able to use *NIP5;1* intron probe signal intensity to infer *NIP5;1* transcriptional activity [55]. For the control gene *PP2A*, median intensities of transcription sites were not significantly affected by B conditions, however *NIP5;1* intron signal intensities were reduced to 46% under 100 μM B compared to the intensities under 0.3 μM B (Fig. 2E). Thus, both smFISH and pre-mRNA qRT-PCR measurements indicate a strong decrease in transcription at high B conditions. However, the observed B-dependent reduction in transcription was smaller than the analytically derived 5-fold reduction based on the observed mRNA accumulation and decay levels. This discrepancy could be due to an incomplete inhibition of transcription by cordycepin, leading to underestimation of the absolute degradation rates under both conditions. The ratio of B-dependent transcription at 0.3 μM B versus 100 μM B (5.3-fold) as was estimated from the mRNA degradation rate and mRNA accumulation (Fig. 1B and D) may therefore overestimate the true transcriptional differences.

### The ATGTAA sequence is required for regulation of B-dependent transcription

**Figure 3.**
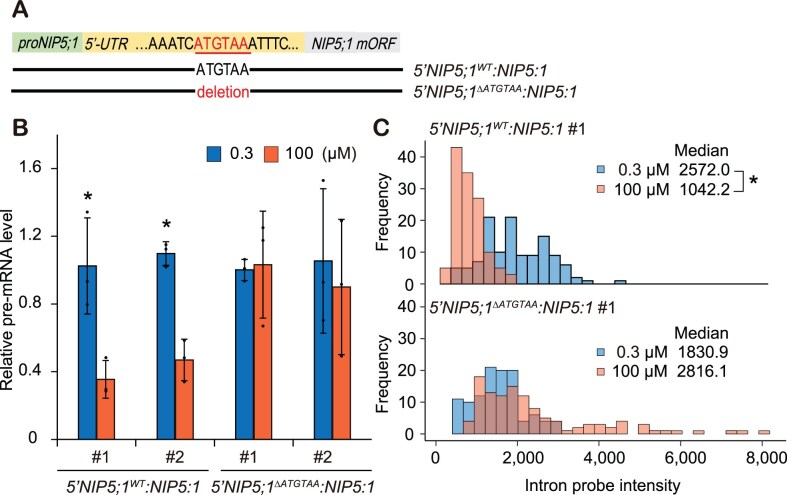
ATGTAA-dependency of *NIP5;1* transcriptional downregulation in response to B. (**A**) Schematic representation of constructs introduced into *nip5;1–1*. (**B**) *NIP5;1* pre-mRNA accumulation. B was shifted to 100 μM B for 2 h after 28-d preculture under 0.3 μM B. pre-mRNA levels were measured by qRT-PCR. Shown are means ± SD (*n* = 4 biological replicates) of relative pre-mRNA accumulation. Asterisks indicate significant difference between B conditions at *P* < .05 using the Student’s *t*-test. (**C**) Transcription activity evaluated by smFISH with intron probe. Histograms represent distribution of *NIP5;1* intron probe intensity of *5′NIP5;1^WT^:NIP5;1* (*n* = 121, 0.3 μM B; *n* = 128, 100 μM B) and *5′NIP5;1^ΔATGTAA^:NIP5;1* (*n* = 112, 0.3 μM B; *n* = 116, 100 μM B). Asterisks indicate significant difference at *P* < .05 using the Wilcoxon rank sum test.

We previously demonstrated that the cauliflower mosaic virus 35S promoter (35S) fused to *NIP5;1* 5′-UTR (*35S*:*NIP5;1 5′UTR*) does not affect B-dependent expression [35], while deleting the ATGTAA sequence in the 5′-UTR abolishes both ribosome stalling and mRNA degradation [8]. We therefore hypothesized that the ATGTAA sequence in the 5′-UTR might also be necessary for the B-dependent transcriptional downregulation. To test this, we evaluated the effect of mutations in ATGTAA on the transcriptional downregulation, through both pre-mRNA measurements and smFISH. *NIP5;1* genomic sequences (from the promoter region to the main ORF’s stop codon) with and without ATGTAA (referred to as *5′NIP5;1^WT^:NIP5;1* and *5′NIP5;1^ΔATGTAA^:NIP5;1*, respectively, Fig. 3A) were introduced into *nip5;1–1*, a knockdown mutant in which *NIP5;1* mRNA accumulation is decreased to ∼1% of the wild-type [8, 28]. Following preculture under 0.3 μM B conditions, these transgenic plants were treated with either 0.3 or 100 μM B for 2 h to allow them to reach steady state. At this point, transgene pre-mRNA levels were measured (Fig. 3B). The shift from 100 μM to 0.3 μM B, resulted in reduced pre-mRNA levels for the *5′NIP5;1^WT^:NIP5;1* line, but not for the 5′-UTR *5′NIP5;1^ΔATGTAA^:NIP5;1* mutant line (Fig. 3B), indicating that the ATGTAA sequence is required for the B-dependent *NIP5;1* transcriptional downregulation. Our smFISH-based transcription activity assessment (Fig. 3C) also revealed B-dependent reduction in fluorescence intensity at the transcription sites in *5′NIP5;1^WT^:NIP5;1* but not in *5′NIP5;1^ΔATGTAA^:NIP5;1*. Together these results from independent experimental approaches support that the ATGTAA sequence is required for B-dependent *NIP5;1* transcriptional downregulation.

### The transcriptional downregulation functions in *trans*

**Figure 4.**
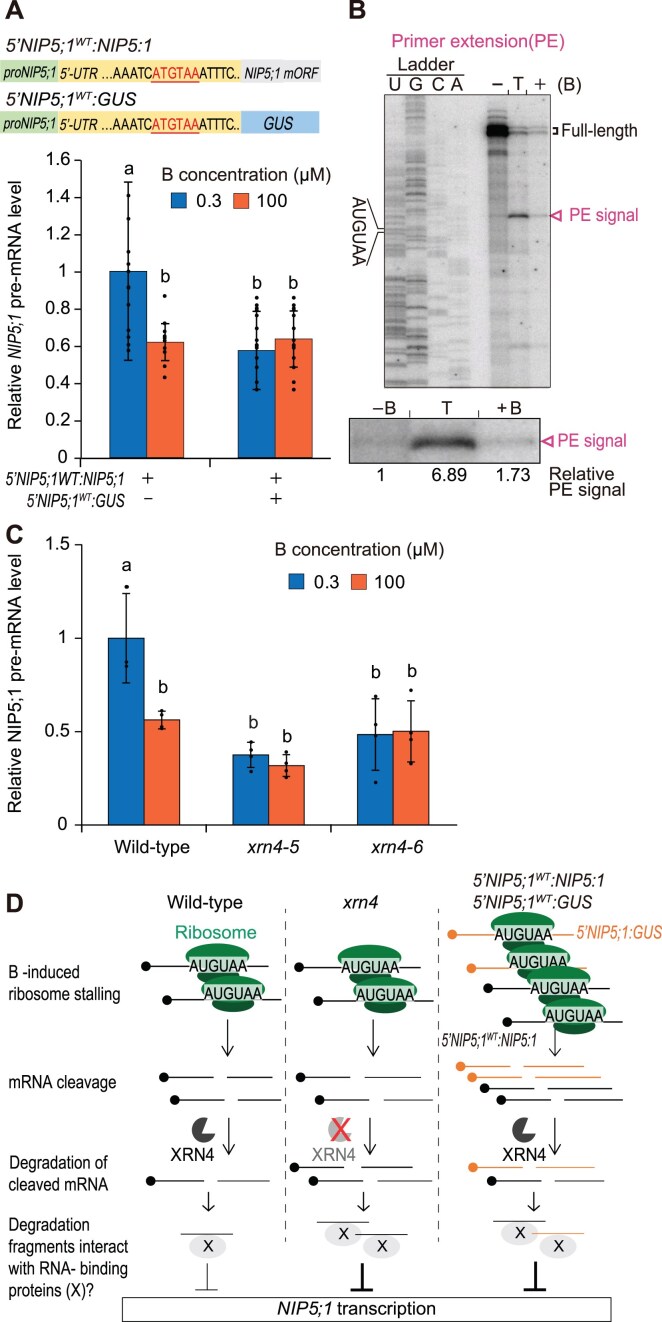
Involvement of mRNA degradation in *NIP5;1* transcriptional downregulation. (**A**) Evaluation of *NIP5;1* transcription activity. *NIP5;1* pre-mRNA was measured by qRT-PCR as an indicator of transcription activity. (top) Schematic representation of constructs. (bottom) Effect of *NIP5;1* 5′-UTR fragments on *NIP5;1* pre-mRNA accumulation. The *5′NIP5;1^WT^:GUS* construct, which harbors *NIP5;1* 5′-UTR but not the coding sequence, was introduced into plants carrying the *5′NIP5;1^WT^:NIP5:1* construct (*n* = 14–17 biological replicates). (**B**) B-dependent *NIP5;1* mRNA degradation intermediates *in vivo*. Wild-type plants were grown for 28 d under 100 μM B (+B) or 0.3 μM B (−B). For the sample in lane T, wild-type plants grown for 28 d under 0.3 μM B were transferred to 100 μM B for 10 min. Poly(A) RNA (500 ng) extracted from *Arabidopsis* roots was used for the primer extension reaction. A sequence ladder was generated using the same primer. The square bracket marks the 5′-end of the full-length mRNA. Primer extension signals corresponding to the 5′-ends of RNA degradation intermediates upstream of AUGUAA are marked with magenta arrowheads to indicate where PE signals are enlarged. Relative primer extension signal intensities are shown. (**C**) Effect of the *xrn4* mutations on *NIP5;1* transcription (*n* = 4 biological replicates). Plants were grown under 10 μM B for 27 d, then transferred to 0.3 μM B for 1 d and treated with 0.3 or 100 μM B for 2 h. Panels (A) and (C) show means ± SD of pre-mRNA accumulation relative to *5′NIP5;1^WT^:NIP5;1* under 0.3 μM B (**A**) or wild-type under 0.3 μM B (**C**); groups sharing the same alphabets are not significantly different at *P* < .05 using the Tukey–Kramer’s test. (**D**) Proposed model for *NIP5;1* transcriptional downregulation. B-dependent transcriptional downregulation of *NIP5;1* is triggered by mRNA degradation products, which result from B-dependent ribosome stalling on AUGUAA in the *NIP5;1* 5′-UTR (wild-type, left). Increasing levels of *NIP5;1* mRNA degradation products in the *xrn4-6* line (middle) or due to the introduction of *NIP5;1* 5′-UTR fragments (right) induces *NIP5;1* transcriptional downregulation. In this model, single sensing event at the translation stage regulates translation, mRNA degradation, and transcription, utilizing mRNA degradation itself as a signal.

Given that ATGTAA is required for transcriptional downregulation, we considered two possible mechanistic models: (i) Ribosome stalling at AUGUAA on the mRNA or subsequent mRNA degradation functions in *trans* as a signal to trigger transcriptional downregulation; or (ii) ATGTAA on the DNA functions as a *cis*-element for the transcriptional downregulation. To distinguish between these two models, we introduced another construct, *NIP5;1* promoter-*NIP5;1*5′-UTR*-GUS* (referred to as *5′NIP5;1^WT^:GUS*), which has already been demonstrated to undergo B-dependent mRNA degradation [35]. This construct was introduced into the transgenic line that carries *5′NIP5;1^WT^:NIP5;1* (Fig. 4A). Because *5′NIP5;1^WT^:GUS* does not harbor the *NIP5;1* main ORF, its introduction could only affect *NIP5;1* pre-mRNA accumulation in *trans*, through transcriptional downregulation of *5′NIP5;1^WT^:NIP5;1*. We found that under 0.3 μM B, introduction of *5′NIP5;1^WT^:GUS* decreased *5′NIP5;1^WT^:NIP5;1* pre-mRNA accumulation to the same level as that of the parental *5′NIP5;1^WT^:NIP5;1* line grown under 100 μM B. This result is consistent with a regulation mechanism functioning in *trans* (Fig. 4A), and inconsistent with it functioning in *cis*. Under 100 μM B, introduction of *5′NIP5;1^WT^:GUS* did not affect *5′NIP5;1^WT^:NIP5;1* pre-mRNA accumulation. Introduction of *5′NIP5;1^WT^:GUS* thus mimics high-B treatment in terms of transcriptional downregulation, considering that *NIP5;1* transcription was already suppressed to its minimum level at 100 μM B. Notably, even a weaker effect of introducing *5′NIP5;1:GUS* would already have suggested a role in trans regulation. The fact that this construct can induce a maximal reduction in transcription was unexpected and will be further explored in the next section. Together with our finding that the ATGTAA sequence is required for transcriptional downregulation, our results suggest that mRNA containing the AUGUAA sequence—rather than the genomic ATGTAA sequence—mediates *NIP5;1* transcriptional downregulation in response to B.

### AUGUAA-mediated *NIP5;1* mRNA degradation fragments are produced even under low B conditions

The observation that introducing *5′NIP5;1^WT^:GUS* downregulates *5′NIP5;1^WT^:NIP5;1* transcription in *trans* implies the involvement of additional factors that mediate information transfer between loci. We hypothesized that mRNA degradation products—specifically, mRNA degradation intermediates—may play a signaling role, such as has previously been proposed for NMD-induced transcriptional regulation [16]. This hypothesis was particularly compelling, as we had previously reported B-dependent accumulation of *NIP5;1* mRNA degradation intermediate fragments with 5′ ends located 13∼14 nt upstream of AUGUAA [8]. To explore this further, we compared absolute abundance of mRNA degradation intermediates at different B conditions by performing primer extension analysis using equal amounts of total RNA extracted from roots (Fig. 4B). A band representing the 5′ ends of mRNA fragments (PE signal) produced by cleavage upstream of AUGUAA was most prominent in samples from plants transferred from 0.3 μM B to 100 μM B (lane T), suggesting a temporal increase in mRNA degradation fragments following B concentration increase. Under steady-state conditions, a stronger signal was observed at 100 μM B (lane ‘+’) compared to 0.3 μM B (lane ‘–’), indicating higher levels of mRNA degradation at elevated B levels. Interestingly, presence of degradation intermediates could even be observed under 0.3 μM B, indicating that a low level of mRNA cleavage takes place even under low B conditions. This can be reasonably explained to be due to the introduction of an additional DNA template of the 5′-UTR within this construct (Fig. 4A), which could elevate mRNA fragment levels under low B to a threshold that triggers transcriptional inhibition.

### Mutation in exonuclease XRN4 downregulates *NIP5;1* transcription

To investigate the role of mRNA degradation intermediates in transcriptional downregulation, we examined the effects of a mutation in *XRN4*, a 5′–3′ exoribonuclease, on *NIP5;1* transcription. The *xrn4* mutation stabilizes *NIP5;1* degradation intermediates [8]. Therefore, if these intermediates are involved in *NIP5;1* transcription downregulation, the *xrn4* mutants should exhibit increased transcriptional repression. Indeed, we observed that regardless of B conditions, the *xrn4* mutant displayed reduced *NIP5;1* pre-mRNA levels, similar to the low levels observed in wild-type plants under high-B conditions (Fig. 4C). This result is consistent with *NIP5;1* transcription in the *xrn4* mutants being suppressed to the minimum level characteristic of the high-B phenotype.

Notably both approaches—introducing *5′NIP5;1^WT^:GUS* and disruption of XRN4 function—yielded similar effects, suggesting that their mechanisms of action on *NIP5;1* transcriptional downregulation are also similar. The *5′NIP5;1^WT^:GUS* experiment indicates the presence of a mobile signal that functions in *trans*, while the impact of the *xrn4* mutation suggests that mRNA degradation intermediate stability influences *NIP5;1* transcription. Taken together, we deduce that mRNA degradation and resultant mRNA fragments are necessary for *NIP5;1* transcriptional downregulation (Fig. 4D).

### sRNA-seq identifies AGO-associated small RNAs potentially derived from *NIP5;1* degradation intermediates

Degradation intermediates of endogenous mRNA that lack either a 5′ cap or a poly(A) tail can be processed into double-stranded RNA (dsRNA) by RNA-dependent RNA polymerase (RDR), leading to the production of small RNAs (sRNAs) [57–59]. To investigate whether *NIP5;1* mRNA degradation intermediates contribute to transcriptional downregulation via an sRNA-mediated mechanism, we examined the potential role of sRNAs in this process. Although sRNAs are traditionally known for their role in RNA interference and mRNA degradation, recent studies have demonstrated their direct involvement in transcriptional regulation as well. One such mechanism involves AGO1–sRNA complexes binding to chromatin to modulate gene expression in response to hormonal cues and stress [44]. While this process typically promotes transcription, which is the opposite to the expected role of sRNAs in *NIP5;1* regulation (where they would be expected to downregulate transcription), we nevertheless explored the potential involvement of AGO1–sRNA complexes in *NIP5;1* transcriptional downregulation.

**Figure 5.**
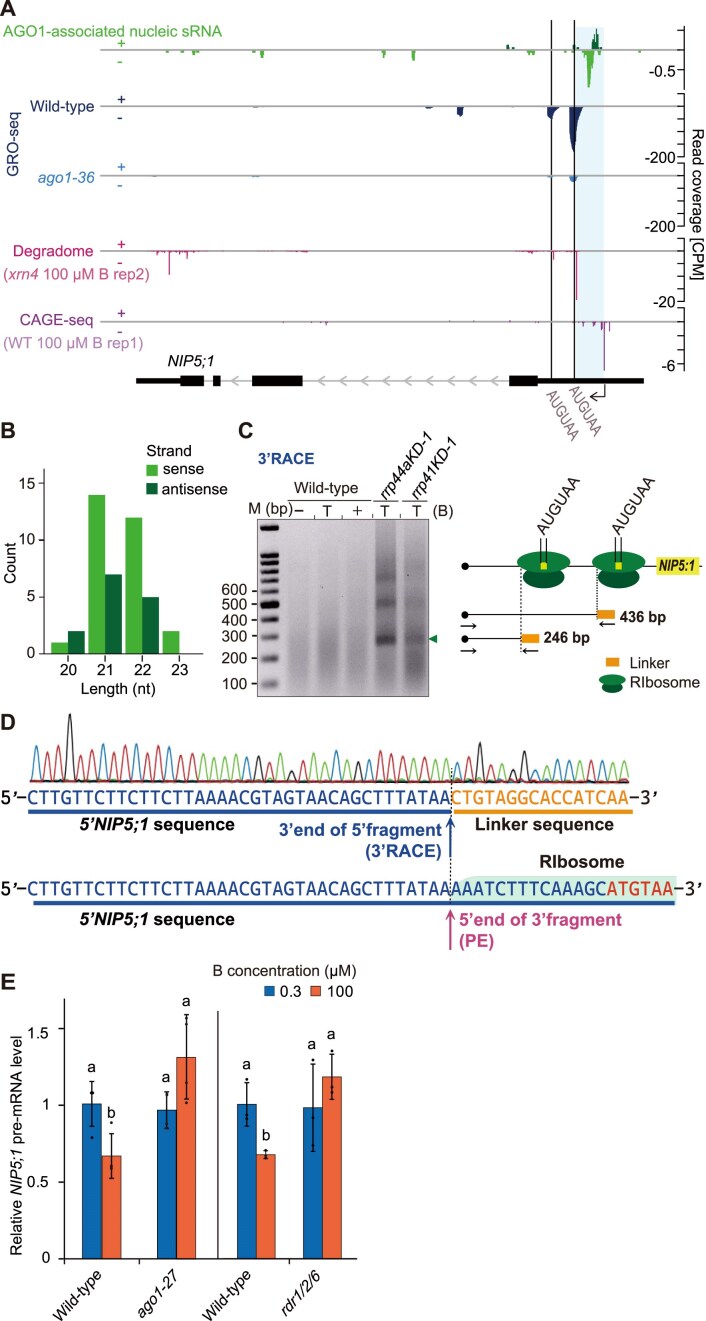
Involvement of AGO1-associated sRNAs in *NIP5;1* transcriptional downregulation. (**A**) Genome browser view of normalized read coverage of GRO-seq. AGO1-associated nucleic sRNA-seq (GSE95301) [44], degradome (GSE119706) [46], and CAGE-seq (PRJDB5794) [45] for *NIP5;1*. SRA datasets were downloaded from NCBI and reanalyzed. Samples are from ten-day-old seedlings grown on 1/2 MS media (50 μM B) [44], two-week seedlings on MS media (100 μM B) [46], and three-day-old seedlings on MS media (100 μM B) [45]. Plus (+) and minus (–) indicate the strandness of the signal. The brown lines indicate the AUGUAA sequence positions. At the bottom, exons (black box), 3 and 5′-UTRs (thin black box) and introns with the direction of transcription (gray arrows) are indicated according to the Araport11 annotation. The 5′ end of capped mRNA detected by CAGE-seq is marked with a black arrow. An expected 5′-degradation fragment denoted by CAGE-seq and degradome signals is highlighted in blue. (**B**) Length of AGO1-associated nucleic sRNAs found upstream of the mRNA cleavage site (highlighted in blue in A). (**C**) The 3′ ends of the 5′ fragments of *NIP5;1* mRNA were identified using 3′ RACE. Plants were grown for 21 d under either 100 μM B (+B) or 0.3 μM B (−B). For the sample in lane T, plants initially grown under 0.3 μM B were transferred to 100 μM B for 10 min. Total RNA (7 μg) was extracted from the roots and used for the 3′ RACE reaction. PCR products obtained from the 3′ RACE reaction were separated on a 1.5% agarose gel. A schematic model shows the expected sizes of the *NIP5;1* mRNA 5′ fragments. (**D**) Sequences obtained after 3′ RACE from the *rrp44a* mutant. The position of the PE, corresponding to the cleavage site of the 5′ end of the 3′ fragments as identified in panel (B) of Fig. 3, is shown. The sequence covered by ribosome stalled on AUGUAA is marked in green. (**E**) Effect of *ago1* and *rdr1/2/6* mutations on *NIP5;1* transcription. Plants were grown under 10 μM B for 27 d, transferred to 0.3 μM B for 1 d, and then treated with 0.3 or 100 μM B for 2 h. pre-mRNA levels of roots were measured by qRT-PCR. Values show mean ± SD (*n* = 4 biological replicates), relative to wild-type, under 0.3 μM B. Groups sharing the same alphabets are not significantly different at *P* < .05 using the Tukey–Kramer’s test.

We first investigated public high-throughput sequencing datasets to explore this potential link. It is important to note that the growth medium (1/2 Murashige and Skoog (MS) medium) typically employed for this type of research contains ∼50 μM B, a concentration at which *NIP5;1* transcription is suppressed to its minimum level (Fig. 1A). Hence, we could only screen for signatures of the downregulation mechanism. First, we assessed an RNA immunoprecipitation sequencing (RIP-seq) dataset that could indicate nuclear AGO1-associated sRNAs [44]. We observed higher levels of 21–22 nt sRNA reads that uniquely map to the sense or antisense strand of the *NIP5;1* 5′-UTR region upstream of the first AUGUAA, compared to other regions of the transcript (Fig. 5A and B). The region enriched with sRNA signals was delimited by the 5′ end of the capped *NIP5;1* mRNA (as denoted by the CAGE-seq peak) [45] and the 5′ end of the *NIP5;1* cleaved mRNA (as denoted by degradome-seq) [46] (Fig. 5A, shaded in blue). This corresponds to the expected region for the 5′ degradation fragments. Given that these sRNA sequences are unique to this region across the entire genome, these results support the hypothesis that they are derived from *NIP5;1* mRNA degradation fragments.

### Endonucleolytic cleavage of *NIP5;1* mRNA produces truncated 5′ fragments

The AGO1-associated sRNA analysis suggested that sRNAs are derived from the 5′ fragments of cleaved *NIP5;1* mRNA. Whereas primer extension and degradome analyses indicated the presence of 3′ fragments downstream of the ribosome stalling site, it remains unclear whether 5′ fragments, which include the sequence corresponding to the sRNAs, are also produced. To address this, we conducted 3′ RACE. Since 3′ fragments of cleaved mRNAs are typically degraded by the 3′–5′ RNA exosome, we used *rrp44a* and *rrp41* mutants which lack RNA catalytic capability [34]. While no band was detected for the wild-type plants, a fragment of around 250 bp was observed for both *rrp44a* and *rrp41* mutants (Fig. 5C). The fragment length corresponded to the 5′ fragment upstream of the ribosome stalling site. Sequencing analysis confirmed that the 3′ end of the 5′ fragment exactly matched the 5′ end of the 3′ fragment detected in the primer extension analysis (Fig. 5D). These results indicate that both the 5′ fragment as well as the 3′ fragment are produced, likely through endonucleolytic cleavage of *NIP5;1* mRNA.

### AGO1 is required for RNA polymerase pausing on *NIP5;1* 5′-UTR

To investigate the role of AGO1 in the *NIP5;1* transcriptional downregulation, we reanalyzed GRO-seq datasets [44]. In wild-type plants, two major peaks were detected in the *NIP5;1* 5′-UTR, located over the two AUGUAA sequences (Fig. 5A). These peaks were sharp, with a diffuse signal observed over other parts of the gene body. These peaks most likely reflect transcriptional pausing rather than active elongation, which would have given rise to a relatively uniform distribution. Notably, these peaks are less pronounced in the *ago1-36* mutant, indicating that AGO1 is involved in transcriptional pausing at these sites.

To further confirm AGO1’s role in *NIP5;1* transcriptional downregulation, we assessed the transcriptional activity of *NIP5;1* in the *ago1* mutant by measuring pre-mRNA accumulation (Fig. 5E). This analysis revealed that B-dependent transcriptional downregulation was completely abolished in the *ago1* mutant, illustrating that AGO1 is essential for B-dependent transcriptional downregulation of *NIP5;1*.

### RDRs are required for B-dependent transcriptional downregulation of *NIP5;1*

The presence of sRNA identical to both the sense and antisense sequences of *NIP5;1* mRNA suggests that dsRNA is synthesized from mRNA degradation intermediates in an RDR-dependent manner (Fig. 5A). To investigate whether B-dependent *NIP5;1* transcriptional downregulation requires RDRs, we measured *NIP5;1* transcriptional activity in the *rdr1-1*/*rdr2-1*/*rdr6-15* (*rdr1/2/6)* triple mutant under both 0.3 and 100 μM B conditions. The triple mutant was used due to uncertainty about which RDR, if any, could be responsible for synthesizing dsRNA from *NIP5:1* mRNA degradation intermediates.

Our analysis of pre-mRNA accumulation revealed that the B-dependent transcriptional downregulation of *NIP5;1* observed in wild-type under high-B conditions was completely abolished in the *rdr1/2/6* mutant (Fig. 5E). This finding indicates that RDRs are essential for B-dependent downregulation of *NIP5;1*. Together with the observation that AGO-associated sRNA matches the *NIP5;1* 5′-UTR, the requirement for both AGO1 and RDRs for B-dependent *NIP5;1* transcriptional downregulation supports an sRNA-mediated mechanism. The model that emerges, is that sRNAs are produced from *NIP5;1* degradation intermediates via the RDR pathway, incorporated into the AGO complex, and subsequently induce RNA Pol II pausing in the *NIP5;1* 5′-UTR-encoding region.

## Discussion

### Functional coupling of mRNA degradation and transcriptional downregulation in *NIP5;1*

Our work reveals that *NIP5;1* gene expression is suppressed through a multifaceted mechanism involving transcriptional downregulation, translational inhibition and mRNA degradation in response to B. Intriguingly, the 5′-UTR AUGUAA sequence plays a key role in all these processes. Our previous work had demonstrated that ribosome stalling at the AUGUAA sequence induces *NIP5;1* mRNA degradation [8]. While we have established that RDRs and AGO1 mediate transcriptional pausing, the exact molecular interactions underlying the coupling of transcriptional downregulation and the ribosome stalling-induced mRNA degradation is as yet not fully characterized. One possible mechanism for AUGUAA-mediated transcriptional downregulation is that the 5′-UTR DNA region containing ATGTAA may function as a transcription factor binding site. In support of this, a recent study demonstrated that the *NIP5;1* genome sequence has two GGNVS motifs within the 5′-UTR that are bound by the STOP1 transcription factor [30]. Interestingly, these two motifs are located just upstream of the two ATGTAA sequences (60 and 52 nucleotides upstream, respectively). Considering that the ATGTAA sequences are not part of the GGNVS motifs, it seems unlikely that these sequences themselves act as binding sites for STOP1. In addition, our observation that the introduction of a construct carrying the 5′-UTR alone (*5′NIP5;1^WT^:GUS*) can downregulate transcription of wild-type *NIP5;1*, supports the involvement of post-transcriptional events in transcriptional downregulation (Fig. 4A). This implies that transcriptional downregulation and ribosome stalling/mRNA degradation are functionally linked, rather than merely sharing the same sequence element.

Although the introduction of *5′NIP5;1^WT^:GUS* affects the transcription of the other transgene, wild-type *NIP5;1*, it does not fully prove that *5*′*NIP5;1^WT^:GUS* was transcribed and translated, nor that ribosome stalling at AUGUAA repressed wild-type *NIP5;1* transcription. Future experiments are needed to determine whether mutations in the promoter or AUGUAA site of *5′NIP5;1^WT^:GUS* indeed affect wild-type *NIP5;1* transcription.

In the mutant *xrn4-6*, which accumulates higher levels of *NIP5;1* mRNA degradation intermediates [8], we observed lower levels of *NIP5;1* transcription under low B conditions compared to wild type plants (Fig. 4C), strongly suggesting that mRNA degradation contributes to *NIP5;1* transcriptional downregulation. However, although we utilized *xrn4* mutants to stabilize mRNA degradation intermediates, we recognize the potential for additional effects of this mutation. XRN1p, a yeast homologue of XRN4, is known to localize to the nucleus in an mRNA degradation-dependent manner, where it binds to certain promoter regions to enhance transcription [18]. If XRN4 has a similar function in *Arabidopsis*, the *xrn4* mutation could suppress *NIP5;1* transcription by eliminating this transcriptional enhancement function rather than through stabilization of mRNA degradation intermediates. However, given that *NIP5;1* transcription in wild type plants is higher under low B conditions when less mRNA degradation is occurring (Figs 4C and 1D), we conclude that it is the stabilization of mRNA degradation intermediates that downregulates *NIP5;1* transcription.

In previously published cases of mRNA degradation-induced transcriptional regulation, such as transcriptional activation by *XRN1p* after mRNA degradation [18] and NMD-induced transcriptional activation [15, 16], the mechanisms serve to compensate for decreased expression levels by upregulating transcription. In contrast, *NIP5;1* presents transcriptional downregulation (as opposed to upregulation), triggered by mRNA degradation in response to high B conditions. This suggests that the underlying mechanism of *NIP5;1* transcriptional regulation is inherently different from those described in these other systems. Notably, in studies of viral infection of human cells, Xrn1-dependent degradation of mRNAs cleaved by viral endonucleases has been demonstrated to trigger the translocation of RNA binding proteins to the nucleus, where they repress RNA Pol II binding to promoters [22]. While we do not rule out the involvement of RNA binding proteins in *NIP5;1* mRNA degradation-dependent transcriptional downregulation, the fact that *NIP5;1* 5′-UTR fragments can downregulate transcription (Fig. 4A), suggest that sequence-specific mechanisms, rather than generalized mRNA cleavage, are primarily responsible for *NIP5;1* regulation. Taken together, our findings indicate that the mechanism of mRNA degradation-dependent transcriptional downregulation of *NIP5;1* is distinct from previously reported processes.

### AGO1-mediated transcriptional downregulation

**Figure 6.**
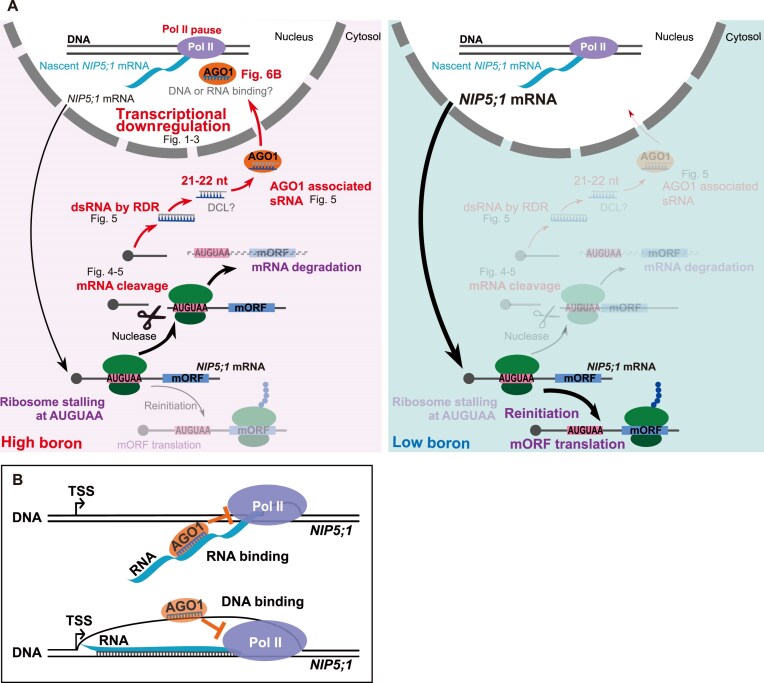
Proposed models for *NIP5;1* transcriptional downregulation. (**A**) Proposed model for the multi-level coordination of the *NIP5;1* response to B conditions. B-dependent ribosome stalling through the AUGUAA sequence results in translational inhibition, mRNA degradation and transcriptional downregulation. Depicted is the proposed process of *NIP5;1* gene expression regulation under both high- (left) and low- (right) B conditions. Black arrows capture the *NIP5;1* pathways, including its translation and degradation. Red arrows represent the process by which parts of the mRNA degradation fragments are incorporated into AGO1, ending up in the nucleus. Ribosome stalling and mRNA degradation (purple) are based on previously published results [8]. Novel processes revealed in this study are shown in red. Hypothetical processes that require further study are highlighted in gray and "?". Transparency indicates low levels of activity for those specific processes. Likewise, arrow thickness indicates the degree of activity. Figure labels within the schematic map the biological processes to the figure(s) in this manuscript containing their supporting evidence. (**B**) Proposed models for the interaction of the AGO1–sRNA complex with either nascent RNA (top) or single-stranded DNA (ssDNA) (bottom). The upper model depicts interaction between nascent RNA and the AGO1–sRNA complex. The AGO1–sRNA complex, which contains antisense sRNAs, binds to nascent RNA transcripts with complementary sequences to the sRNA. This interaction disrupts Pol II elongation, leading to transcriptional inhibition. The lower model depicts interaction between ssDNA and the AGO1–sRNA complex through the R-loop structure. In this case, the AGO1–sRNA complex, carrying sense-strand sRNAs, binds to the exposed ssDNA, resulting in the inhibition of Pol II elongation. TSS, transcription start site.

The AGO1-associated sRNA-seq data indicated that sRNAs mapping to the *NIP5;1* 5′-UTR form complexes with AGO1 in the nucleus. Notably, neither GRO-seq nor CAGE-seq detected any transcriptional signal from the *NIP5;1* antisense strand (i.e. the genome plus strand) (Fig. 5A). It is therefore unlikely that the detected antisense sRNAs are transcribed from the genomic DNA. To our knowledge, the only known pathway capable of producing antisense sRNAs identical to the *NIP5;1* mRNA is RDR-dependent dsRNA synthesis from mRNA degradation fragments [57–59]. Given that mutations in the RDRs abolished B-dependent transcriptional downregulation of *NIP5;1* (Fig. 5E), it is likely that the observed antisense sRNAs are synthesized via a pathway similar to the conventional siRNA pathway, originating from mRNA degradation intermediates. These intermediates are converted into precursor dsRNA, processed into 21–22 nt small dsRNAs by DICER-LIKE (DCL) proteins, and subsequently incorporated into AGO1 (Fig. 6A).

This model is supported by recent studies showing that dsRNA production triggered by ribosome stalling-induced mRNA degradation occurs during the generation of siRNA from transposon-derived RNAs [60, 61] and during trans-acting siRNA (tasi-RNA) biogenesis [62, 63]. Furthermore, truncated mRNA lacking poly(A) tails, such as the 5′ fragment of *NIP5;1* mRNA after cleavage, has been shown to be more prone to RDR-mediated dsRNA synthesis [63–65]. RNA silencing is thought to occur when RDR substrate levels exceed a certain threshold, with these levels influenced by RNA decay pathways such as exonucleolytic decay [66]. In our study, however, *NIP5;1* transcriptional inhibition was not observed for all the concentrations at which mRNA degradation was detected. Specifically, steady state mRNA levels, but not pre-mRNA levels, were reduced at 10 μM B compared to 0.3 μM B, supporting that mRNA decay but not transcriptional repression was triggered at this concentration (Fig. 1B and E). This observation aligns with the threshold model of siRNA production, where increased abundance of RDR substrate (mRNA degradation intermediates) under higher B concentrations accounts for the observed transcriptional repression. However, we do not exclude the possibility of additional transcription repression pathways independent of mRNA decay, which should be investigated in future studies.

In the GRO-seq profile, two AGO1-dependent Pol II pausing signals were observed downstream of the sRNA signals (Fig. 5A), pointing to interactions between the AGO1–sRNA complex and the transcriptional machinery. There are two major possibilities for how this interaction might occur, depending on whether the AGO1–sRNA complexes bind to the nascent mRNA or to the genomic DNA.

The first possibility is that the AGO1–sRNA complexes bind directly to nascent *NIP5;1* mRNA, affecting Pol II progression (Fig. 6B upper model). In the conventional siRNA pathway, AGO1 bound to 21-nt sRNA induces mRNA cleavage, but such complexes are generally unstable. However, AGO1 bound to 22-nt sRNA is known to form relatively stable complexes with mRNA [64]. The majority of the AGO1-associated sRNAs homologous to *NIP5;1* 5′-UTR sequences were either 21 or 22 nt in length (Fig. 5B), and AGO1 bound to those 22 nt sRNAs could thus form stable complexes with the nascent *NIP5;1* mRNA. Those generated complexes could then modulate transcription, comparable to the way in which AGO homolog NRDE-3 is acting in *C. elegans*: NRDE-3 binds to nascent mRNA via its complementary siRNA, recruits NRDE-2 to form a complex, which then inhibits Pol II elongation to repress transcription [67]. Although AGO1–sRNA binding to nascent *NIP5;1* RNA could potentially also result in pre-mRNA cleavage, instead of Pol II pausing, this scenario seems unlikely, given the observation that B conditions do not significantly affect the half-life of *NIP5;1* pre-mRNA (Supplementary Fig. S2).

An alternative model is that the AGO1–sRNA complexes bind to genomic DNA corresponding to the *NIP5;1* 5′-UTR region, thereby directly inhibiting Pol II progression (Fig. 6B lower panel). Although AGO1–sRNA complexes primarily bind complementary RNA, they have been shown *in vitro* to bind ssDNA as well, albeit with lower affinity [68]. For this model to hold, the target DNA region must be single stranded. During Pol II progression, ∼10 base pairs of DNA are temporarily unwound and reannealed into dsDNA after Pol II passes [69]. It seems unlikely that AGO-sRNA would bind to this short moving window of ssDNA. However, longer ssDNA regions may arise during transcription, particularly when the nascent RNA hybridizes to the anti-sense DNA strand, preventing DNA reannealing. These DNA/RNA hybrid structures, known as R-loops, are estimated to form in ∼10% of the *Arabidopsis* genome [70]. Indeed, in a previous study, R-loop formation was detected in the *NIP5;1* 5′-UTR region [71]. Therefore, AGO1–sRNA may potentially bind during transcription to the ssDNA region of the *NIP5;1* gene to inhibit Pol II progression. Although AGO1–sRNA binding to DNA has not been demonstrated *in vivo*, and there is no direct evidence supporting this model, the presence of R-loops in the *NIP5;1* 5′-UTR region renders this an intriguing possibility.

### Transient acceleration of *NIP5;1* mRNA degradation and transcriptional downregulation in response to B

The rate of *NIP5;1* mRNA degradation exhibits a transient increase following exposure to elevated B concentrations (Fig. 1C and D). Upon transfer to a high B environment, *NIP5;1* mRNA degradation accelerates sharply reaching a peak value after five minutes, at which the degradation rate is 28-fold increased. This is followed by a slow decrease in the degradation rate, stabilizing after ∼120 min. Although this steady state rate is 5.7-fold lower than the peak rate, it is still 4.7-fold higher than the pre-treatment rate. This transient surge in degradation likely serves to rapidly eliminate pre-existing *NIP5;1* mRNA that encodes a B transporter that becomes detrimental under high B conditions. Moreover, the swiftness of the transporter regulation is particularly critical for maintaining a stable nutrient flow across multiple cell layers, a universal requirement for multicellular directional-transport systems in which substrate levels directly influence transporter expression [39].

In general, transcription rate reduction is a strategy to save energy especially when the protein might be not needed over longer time-scales. However, while *NIP5;1* transcription is indeed downregulated under high-B conditions, we have observed that it does not cease entirely. Instead, a low but sustained level of mRNA is maintained alongside an increased degradation rate. At first glance, this regulation system may therefore appear inefficient. However, if mRNA degradation serves as a signal for transcriptional downregulation, then a continuous supply of *NIP5;1* transcripts would be required to sustain this regulatory feed-back loop. This suggests that even under high-B conditions, a certain level of *NIP5;1* mRNA may be necessary, not for protein production, but for sensing environmental B levels.

In our model, mRNA degradation fragments act as molecular signals generated during translation in a B-dependent manner (Fig. 6A). The rate at which these degradation products accumulate should therefore reflect both the ongoing protein production rate and the cytosolic B concentration. This mechanism enables the plant to continuously monitor and adjust *NIP5;*1 expression in response to dynamic environmental conditions. Moreover, this type of feedback regulation likely provides an evolutionary advantage by repurposing degradation byproducts as signaling molecules, circumventing the need for additional dedicated signaling factors. The widespread involvement of RNA binding proteins in diverse cellular processes, including chromatin modification [72] further supports this concept.

In conclusion, we demonstrate that B-dependent transcriptional downregulation of *NIP5;1* requires the 5′-UTR ATGTAA sequence—the same sequence element responsible for B-dependent ribosome stalling and subsequent mRNA degradation. Additionally, we show that AGO1 and mRNA degradation products play a critical role in this regulatory process. Together, these findings support a dynamic model in which mRNA degradation and resulting decay intermediates function as signals for transcriptional downregulation. In our model, B-dependent ribosome stalling functions as the core integrative sensing mechanism, coordinating regulation at multiple different levels: transcription, translation, and mRNA degradation. This study reveals a unique feedback mechanism linking mRNA degradation to transcriptional control. It represents a broader regulatory strategy that may extend beyond plants to other organisms adapting to dynamic environmental conditions or facing tissue-level dynamical constraints.

## Supplementary Material

gkaf159_Supplemental_File

## Data Availability

The data underlying this article are available in the article and in its online supplementary material.
