# Supplementary material for: Ribosome stalling-induced NIP5;1 mRNA decay triggers ARGONAUTE1-dependent transcription downregulation
Source: Nucleic Acids Res. 2025 Mar 20;53(5):gkaf159. doi: 10.1093/nar/gkaf159 (PMC11915504; doi:10.1093/nar/gkaf159)
Supplement: gkaf159_Supplemental_File [file gkaf159_supplemental_file.pdf]

Supplementary Information

Ribosome stalling-induced *NIP5;1* mRNA decay triggers ARGONAUTE1-dependent transcription down-regulation

Mayuki Tanaka[1,2], Naoyuki Sotta[1,2,3,*], Susan Duncan[4], Yukako Chiba[5,6], Hitoshi Onouchi[7], Athanasius F. M. Marée[3,*], Satoshi Naito[5,7], Verônica A. Grieneisen[3,*], Toru Fujiwara[1,*]

[1]Graduate School of Agricultural and Life Sciences, The University of Tokyo, Tokyo 113-8657, Japan.
[2]Graduate School of Agriculture, Osaka Metropolitan University, Osaka 599-8531, Japan.
[3]School of Biosciences, Cardiff University, Cardiff, CF10 3AX, United Kingdom.
[4]Department of Cell and Developmental Biology, John Innes Centre, Norwich Research Park, Norwich, NR4 7UH, United Kingdom.
[5]Graduate School of Life Science, Hokkaido University, Sapporo 060-0810, Japan.
[6]Faculty of Science, Hokkaido University, Sapporo 060-0810, Japan.
[7]Graduate School of Agriculture, Hokkaido University, Sapporo 060-8589, Japan.
[*] To whom correspondence should be addressed. Tel: +81 (0)3-5841-5104; E - mail: atorufu@g.ecc.u-tokyo.ac.jp
Correspondence may also be addressed to Naoyuki Sotta. Email: nsotta@omu.ac.jp, Athanasius F. M. Marée. Email: marees@cardiff.ac.uk, Verônica A. Grieneisen. Email: GrieneisenV@cardiff.ac.uk

Contents

Supplementary Figures S1 – S2
Supplementary Table S1

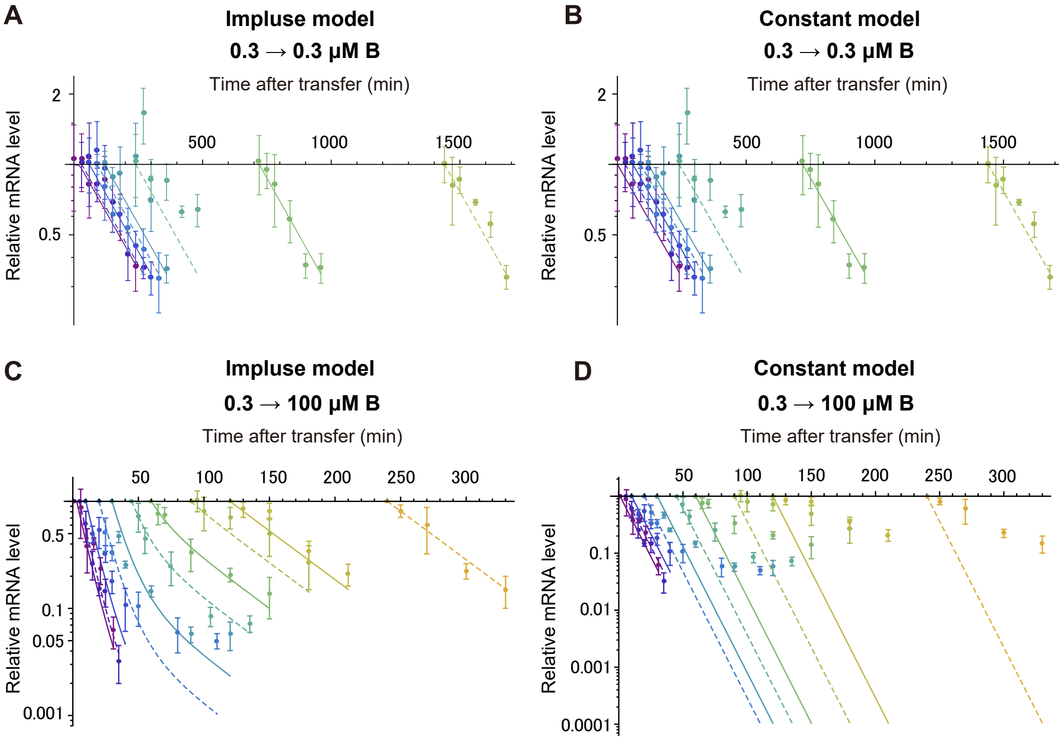

E

B concentration (μM)	Impulse model	Constant model
0.3 → 0.3	- 509.2	- 517.1
0.3 →100	- 468.5	- 209.1

Supplementary Figure S1. Time-dependent *NIP5;1* mRNA degradation in wild-type. Plants grown under 0.3 μM B for 28 days and transferred to medium containing 100 (**A** and **B**) and 0.3 (**C** and **D**) μM B conditions for 1 to 240 min, after which cordycepin, an inhibitor of transcription, was applied. Root samples were harvested 0 to 120 min after cordycepin application. *NIP5;1* mRNA levels were measured by RT-qPCR. mRNA levels after cordycepin application were normalized to those at t=0 (n = 3-4 biological replicates). Impluse model (**A** and **C**) and constant model (**B** and **D**) are shown. (**E**) Akaike's information criteria (AICc) values for each model.

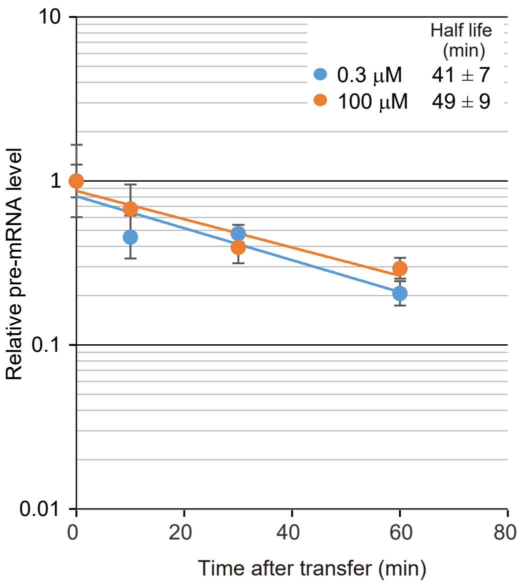

Supplementary Figure S2. ***NIP5;1*** **pre-mRNA degradation rates at the steady-state levels.**

Wild-type plants were grown under 0.3 or 100 μM B conditions for 27 days and were then transferred to medium containing 0.3 μM B or 100 μM B, respectively, for 1 day, after which cordycepin was applied. Root samples were harvested from 0 to 60 min after cordycepin application, and *NIP5;1* pre-mRNA levels were measured by RT-qPCR. pre-mRNA levels after cordycepin application were normalized to those at t = 0. Means ± SD of relative mRNA accumulation (n = 4 biological replicates) are shown. *NIP5;1* pre-mRNA half-lives were determined by linear regression of log-converted relative mRNA amounts.

Sipplementary Table S1. Public datasets used in this study.

Experiment type	Sample	Sample name	Series	Reference
GRO-seq	GSM2522257	GROseq_ago1-36_rep1	GSE95301	1
	GSM2522258	GROseq_ago1-36_rep2		
	GSM2522259	GROseq_WT_rep1		
	GSM2522260	GROseq_WT_rep2		
AGO1-associated nucleic small RNA-seq	GSM2522278	sRNAseq_NuclAGO1_WT_JAcontrol		
CAGE-seq	SAMD00080633	CAGE data of wild type under dark condition (control)	PRJDB5794	2
degradome-seq	GSM3381086	ATH362 dataset 2	GSE119706	3

1. Liu et al, Arabidopsis ARGONAUTE 1 binds chromatin to promote gene transcription in response to hormones and stresses. Dev. Cell 44, 348-361. e7. 10 (2018).

2. Y. Kurihara et al., Transcripts from downstream alternative transcription start sites evade uORF-mediated inhibition of gene expression in arabidopsis. Proc. Natl. Acad. Sci. USA. 115, 7831-7836 (2018).

3. V.K. Nagarajan, P.M. Kukulich, B. von Hagel, P.J. Green, RNA degradomes reveal substrates and importance for dark and nitrogen stress responses of arabidopsis XRN4. Nucleic Acids Res. 47 9216-9230 (2019).
